# Dietary lipids in glycogen storage disease type III: A systematic literature study, case studies, and future recommendations

**DOI:** 10.1002/jimd.12224

**Published:** 2020-02-26

**Authors:** Alessandro Rossi, Irene J. Hoogeveen, Vanessa B. Bastek, Foekje de Boer, Chiara Montanari, Uta Meyer, Arianna Maiorana, Andrea Bordugo, Alice Dianin, Carmen Campana, Miriam Rigoldi, Priya S. Kishnani, Surekha Pendyal, Pietro Strisciuglio, Serena Gasperini, Giancarlo Parenti, Rossella Parini, Sabrina Paci, Daniela Melis, Terry G. J. Derks

**Affiliations:** ^1^ Department of Translational Medicine, Section of Pediatrics University of Naples “Federico II” Naples Italy; ^2^ Section of Metabolic Diseases Beatrix Children's Hospital, University Medical Center Groningen, University of Groningen Groningen The Netherlands; ^3^ Department of Pediatrics San Paolo Hospital, ASST Santi Paolo e Carlo, University of Milan Milan Italy; ^4^ Department of Pediatrics Hannover Medical School Hannover Germany; ^5^ Division of Metabolic Diseases, Department of Pediatric Specialties Bambino Gesù Children's Hospital Rome Italy; ^6^ Inherited Metabolic Diseases Unit, Department of Paediatrics, Regional Centre for Newborn Screening, Diagnosis and Treatment of Inherited Metabolic Diseases and Congenital Endocrine Diseases Azienda Ospedaliera Universitaria Integrata Verona Italy; ^7^ Rare Diseases Center ASST Monza, San Gerardo Hospital Monza Italy; ^8^ Division of Medical Genetics, Department of Pediatrics Duke University Medical Center Durham North Carolina USA; ^9^ Rare Metabolic Diseases Pediatric Center, Pediatric Clinic, Fondazione MBBM, San Gerardo Hospital Monza Italy; ^10^ Department of Medicine, Surgery and Dentistry "Scuola Medica Salernitana" Section of Pediatrics, University of Salerno Baronissi (SA) Italy

**Keywords:** dietary intervention, glycogen storage diseases, high fat, medium‐chain triglycerides, metabolic control

## Abstract

A potential role of dietary lipids in the management of hepatic glycogen storage diseases (GSDs) has been proposed, but no consensus on management guidelines exists. The aim of this study was to describe current experiences with dietary lipid manipulations in hepatic GSD patients. An international study was set up to identify published and unpublished cases describing hepatic GSD patients with a dietary lipid manipulation. A literature search was performed according to the Cochrane Collaboration methodology through PubMed and EMBASE (up to December 2018). All delegates who attended the dietetics session at the IGSD2017, Groningen were invited to share unpublished cases. Due to multiple biases, only data on GSDIII were presented. A total of 28 cases with GSDIII and a dietary lipid manipulation were identified. Main indications were cardiomyopathy and/or myopathy. A high fat diet was the most common dietary lipid manipulation. A decline in creatine kinase concentrations (*n* = 19, *P* < .001) and a decrease in cardiac hypertrophy in paediatric GSDIIIa patients (*n* = 7, *P* < .01) were observed after the introduction with a high fat diet. This study presents an international cohort of GSDIII patients with different dietary lipid manipulations. High fat diet may be beneficial in paediatric GSDIIIa patients with cardiac hypertrophy, but careful long‐term monitoring for potential complications is warranted, such as growth restriction, liver inflammation, and hepatocellular carcinoma development.

**Abbreviations**
CK: creatine kinase
E‐%: energy percentage of total caloric intake
GSD: glycogen storage disease
IVSd: interventricular septum dimension
MCT: medium‐chain triglycerides
TG: triglycerides

## 1 INTRODUCTION

Glycogen storage diseases (GSD) are inborn errors of glycogen synthesis or degradation. Although a wide spectrum of clinical and biochemical presentation is observed, GSD are usually classified into hepatic and muscle GSD. Primary manifestations of the hepatic GSD subtypes 0, I, III, VI, IX, and XI are fasting intolerance‐associated hypoglycaemia, hepatomegaly and failure to thrive. In addition, GSDIII patients also show a myopathic phenotype with skeletal muscle involvement and/or cardiomyopathy.^1^

Management guidelines have been published for GSD subtypes Ia,^2^, ^3^ Ib,^4^ III,^5^ and VI and IX together.^6^ Dietary management is the cornerstone of treatment for hepatic GSD patients to maintain normoglycaemia, prevent secondary metabolic derangements and long‐term complications. Strict dietary management and compliance has significantly improved the outcomes for many GSD patients.^7^, ^8^ Traditionally, dietary carbohydrates and protein have received most interest, whereas lipids usually have been restricted. Several case reports have described beneficial effects of dietary lipid manipulations in hepatic GSD patients, including (modified) ketogenic diets and medium‐chain triglyceride (MCT) enrichment.^9^, ^10^, ^11^, ^12^, ^13^ However, the role of dietary lipids as a third macronutrient in dietary management is still controversial.^14^

The aim of this study was to describe current experiences with dietary lipid manipulations in hepatic GSD patients. We performed a systematic literature study of all published cases describing hepatic GSD patients with a dietary lipid manipulation. Thereafter, an international, observational, retrospective study was performed to include unpublished cases. The subsequent discussion provides recommendations for future patient care and research.

## 2 METHODS

### 2.1 Systematic literature study

Published cases were retrieved by a systematic literature search conducted according to the Cochrane Collaboration methodology on December 31, 2018. PubMed and EMBASE were searched using both MeSH terms and free text. A flowchart of the detailed search strategy can be found in Supplementary File S1. Initially, all hepatic GSD patients with a dietary lipid manipulation were identified. However, the majority of cases describing GSD type I and VI patients were published before the introduction of management guidelines and lacked important clinical information.^15^, ^16^, ^17^, ^18^ Therefore, these data were not included, and further data analysis was solely focused on GSDIII. All reports about GSDIII patients receiving dietary lipid manipulation were included. Inclusion criteria were GSDIII diagnosis based on biochemical or molecular evaluation and English language. Exclusion criteria were no individual data presentation and/or absence of follow‐up data. Two independent reviewers (I.J.H., V.B.B.) performed title, abstract screening and subsequently full‐text assessment. After selection of eligible full‐text papers and conference abstracts, case information was collected in a data table specifically designed for the purpose of this study, including patient's age at start dietary intervention, gender, GSDIII subtype, indication to start dietary intervention, specifications of diet, duration of the intervention and follow‐up, and outcome measures (laboratory results, imaging tests, and clinical picture).

### 2.2 Case studies

Unpublished cases were retrieved via the International GSD Conference 2017, organised in Groningen, The Netherlands on June 15 to 17, 2017. All metabolic dieticians were invited to join a networking session on the role of MCT in hepatic GSD. In October 2017, after the IGSD2017, all delegates who had attended the networking session received an invitation by email to share unpublished data of hepatic GSD patients with a dietary lipid manipulation. Data were collected through the same table used for published cases.

### 2.3 Data synthesis and analysis

Data on macronutrients were presented as energy percentage (E‐%) of total caloric intake, or if otherwise noted in the legend. MCT supplementation was defined as regular GSD diet enriched in MCT. MCT replacement was defined as long‐chain triglycerides substituted with MCT. High fat diet was defined as a diet in which lipids were the main macronutrient based on E‐% values. Ketogenic diets were also categorised as high fat even in the absence of E‐% values. Standard deviations of BMI were calculated using standard growth charts established by the CDC/2000. Age specific outcomes were presented as Z‐scores or in subgroups (ie, child and adult). The cutoff value for adulthood was set at 16 years of age. Laboratory parameters were presented as range (minimum‐maximum value) before and after the dietary intervention, respectively. For each parameter, individual differences (Δ) were presented as percentage difference between mean values before and after the dietary intervention, respectively. Concentrations were considered increased when Δ > +10%, decreased when Δ < −10% and stable if Δ between −10% and +10%. Z‐scores were calculated for interventricular septum dimensions (IVSd) to normalise for the body surface area. For Z‐score calculation, the regression equation by Pettersen was used.^19^ The Haycock formula was used for BSA calculation.^20^

### 2.4 Statistical analysis

Data were analysed using Prism 7 software (GraphPad Software, Inc. La Jolla, California) and Statistical Package for Social Sciences, version 23.0 (SPSS, IBM Corp., Armonk, New York). Differences in outcome measures before and after dietary lipid manipulation were analysed with a paired *t* test if data were normally distributed (assessed by the Shapiro‐Wilk test). Data were analysed with Wilcoxon signed ranks test in case of non‐normally distributed data after log‐transformation. Pearson's or Spearman's correlations tests were used to define relationships between dietary parameters and changes in laboratory outcomes. Statistical significance was defined as *P* < .05.

## 3 RESULTS

### 3.1 Cases

Literature search revealed four full text articles and five conference abstracts describing 14 GSDIII patients (Supplementary File S2), whereas 14 unpublished cases were collected from six metabolic centres from three different countries (Supplementary File S3). Therefore, a total of 28 cases with GSDIII and a dietary lipid manipulation were collected.

### 3.2 Patients features, indication to start the diet and compliance

Main features of GSDIII patients receiving a dietary lipid manipulation are presented in Table 1. The main indication to start the dietary intervention was cardiomyopathy and/or myopathy. Four patients (cases 9, 19, 26, 27) did not follow the modified diet regimen regularly: either poor compliance was reported, or the diet was discontinued several times.

**Table 1:** Features of published and unpublished cases with GSDIII and a dietary lipid manipulation (*n* = 28)

Cases, *n*	
Published	14
Unpublished	14
Total	28
*Gender*, *n* (%)	
Male	11 (39%)
Female	15 (54%)
Unknown	2 (7%)
*Age* a (y)	
Median [range]	7 [0‐41]
*Indication*, *n* (%)	
Hyperlipidaemia	2 (7%)
Poor metabolic control	7 (25%)
Muscle involvement	19 (68%)
Skeletal muscle weakness	3
Cardiomyopathy	6
Skeletal and cardiac muscle involvement	9
Hypotonia	1
*Intervention*, *n* (%)	
High fat diet	26^b^ (93%)
MCT supplementation/replacement	6 (21%)
Atkins, ketogenic diet	5 (18%)
Corn oil supplementation	1 (4%)
*Months of dietary intervention*	
Median [range]	18 [1‐60]

a Age at start dietary intervention.

b Four patients received both MCT and a high fat diet (cases 15, 16, 20, and 21), five patients received a ketogenic diet which was also categorised as high fat diet (cases 2 and 8‐11), one patient received a high fat diet with corn oil substitution (case 14),^17^ and one patient received a high fat diet supplemented with d,l‐3‐hydroxybutyrate (case 12).^13^

Abbreviation: MCT, medium‐chain triglyceride.

### 3.3 Diet composition

**Figure 1:**
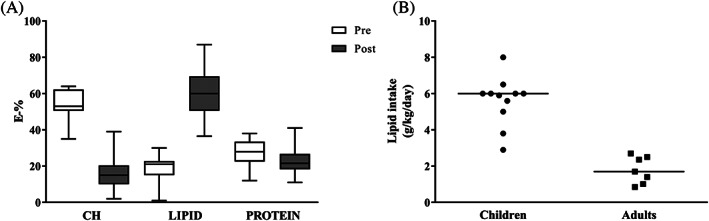
Dietary features of GSDIII patients. A, Diet composition in GSDIII patients before (*n* = 10) and after (*n* = 24) high fat diet. B, Lipid intake in GSDIII patients receiving high fat diet (*n* = 18, patients on high fat diet also receiving MCT supplementation were included). Data are presented as median [range]. CH, carbohydrates

Most common lipid manipulation was high fat diet (Table 1). Figure 1A presents the diet composition before and after dietary intervention in GSDIII patients receiving a high fat diet. Lipid intake ranged from 0.9 to 8.0 g/kg/day (2.9‐8.0 g/kg/day in children, 0.9‐2.7 g/kg/day in adults) (Figure 1B).

Less common interventions included corn oil supplementation together with high fat diet (case 14),^17^ and MCT supplementation alone (cases 6 and 7)^21^ (Supplementary File S2).

### 3.4 Laboratory results

**Figure 2:**
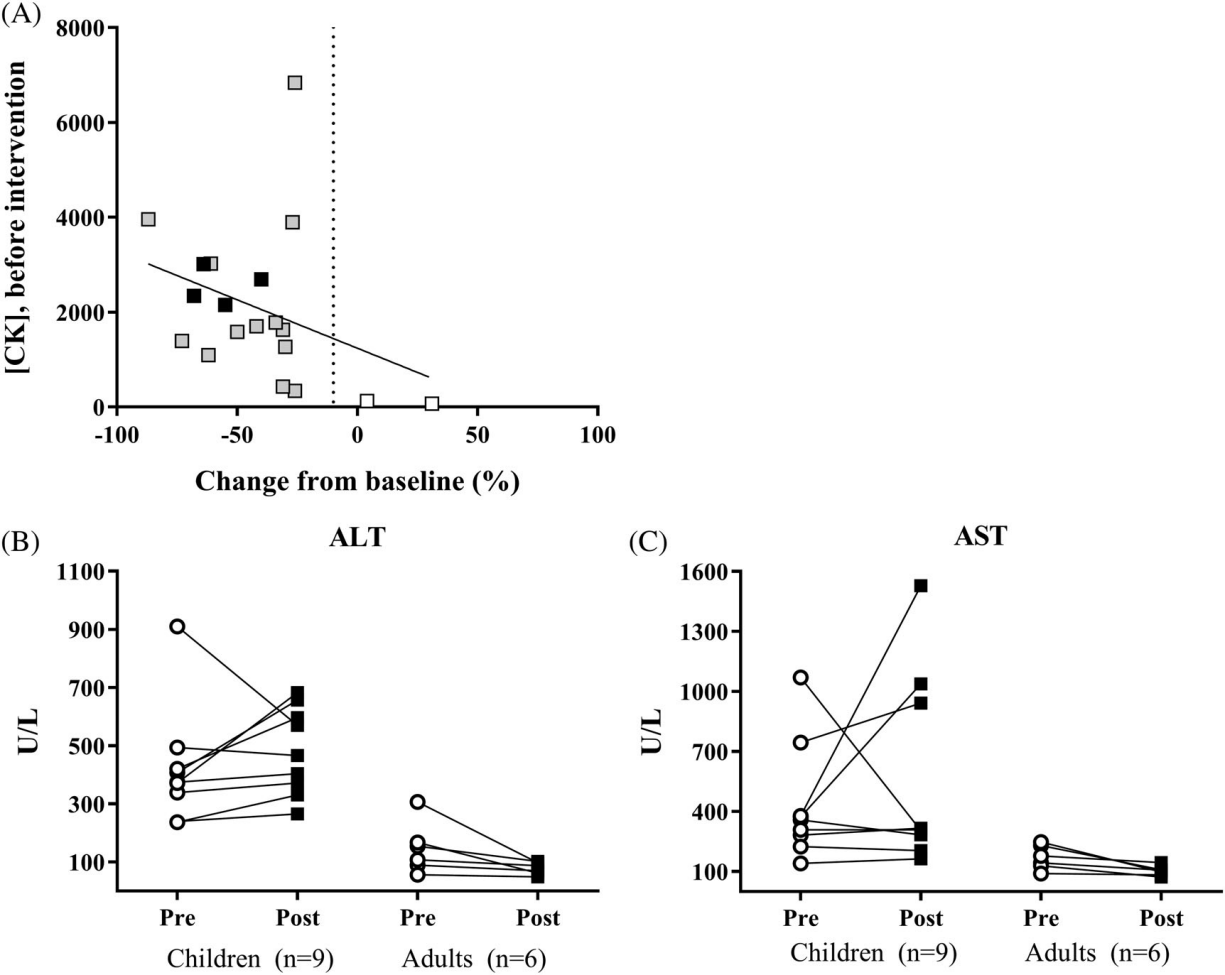
Changes in laboratory parameters by dietary lipid manipulation in GSDIII. A, Relation between CK concentrations before intervention and change in CK concentration of 19 individual patients with GSDIII with high fat diet, including patients with combined high fat diet and MCT supplementation (*n* = 4). Spearman's rho correlation coefficient = −0.40, *P* > .05. Grey square; GSDIII patient, black square; GSDIII patient receiving combined high fat diet and MCT supplementation, white square; GSDIII patient showing CK concentrations within age‐related reference values before and after dietary lipid manipulation.^22^ B, Measured blood ALT concentrations in GSDIII patients before (circle) and after (square) the introduction of a high fat diet. C, Measured blood AST concentrations in GSDIII patients before (circle) and after (square) the introduction of a high fat diet

The changes in laboratory parameters in GSDIII patients receiving high fat diet are presented in Figure 2 and Supplementary File S4.

Creatine kinase (CK) concentrations were available in 73% (19/26) of GSDIII patients receiving high fat diet (Figure 2A). Mean CK concentrations were lower after receiving high fat diet in 89% (17/19) of GSDIII patients (2070 U/L ± 1634 vs 1078 U/L ± 1148, *P* < .001). One previously unreported patient showed an increase in CK concentrations (case 25); however, CK concentrations remained within the reference range.^22^ Another patient showed stable CK concentrations (case 26). No correlations between ΔCK and changes in macronutrients were found.

Liver transaminases (ALT/AST) were documented in 58% (15/26) of GSDIII patients on a high fat diet (Figure 2B,C). In adult GSDIII patients, ALT concentrations decreased in all cases (*n* = 6); AST concentrations decreased in five patients (83%) and were stable in the sixth patient. In paediatric GSDIII patients, ALT concentrations increased in four patients (44%), decreased in one patient (11%) and were stable in four patients (44%); AST concentrations increased in five patients (56%), decreased in two patients (22%), and were stable in two patients (22%).

### 3.5 Imaging and clinical outcomes

**Figure 3:**
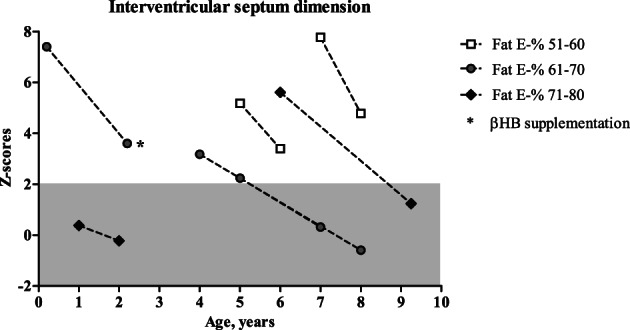
Effect of high fat diet on interventricular septum dimension in paediatric GSDIIIa patients (*n* = 7). Measurements are displayed as Z‐scores. GSDIIIa subjects are noted with symbols according to E‐% of fat. Grey column represents range of normal Z‐scores

IVSd Z‐scores decreased in paediatric GSDIII patients with a high fat diet (*n* = 7, *P* < .01; Figure 3), but not in adult GSDIII patients (*n* = 4, Supplementary File S3). There were no correlations between the change in IVSd Z‐scores and changes in macronutrients. Data on muscle ultrasound and muscle function tests were available in two adult GSDIIIa patients on a high fat diet with MCT replacement (cases 15 and 16). There was no effect on muscle density. Muscle strength as assessed with dynamometry improved only for case 15. Subjective improvements of exercise tolerance and/or muscle strength were reported in 78% (14/18) of paediatric GSDIII patients and 50% (4/8) of adult GSDIII patients on high fat diet.

Among paediatric GSDIII patients receiving a high fat diet 18% (2/11) showed improved height SDS, 64% (7/11) showed stable height SDS and 18% (2/11) showed decreased height SDS. All paediatric patients showed normal BMI (60% stable, 40% normalised). BMI was stable in all adult GSDIII patients.

### 3.6 Side effects

Side effects were reported in two patients. Hypoglycaemia is an intrinsic symptom of hepatic GSD and was reported in two GSDIII patients on a high fat diet. Specifically, one paediatric GSDIIIa patient (case 18) reported isolated hypoglycaemia 3 years after the start of a high fat diet, and one paediatric GSDIIIa patient (case 19) presented with an isolated hypoglycaemia 1 year before and 2 years after starting with a high fat diet.

## 4 DISCUSSION

Complex carbohydrates and, for ketotic GSD patients, protein enrichment are the cornerstones of dietary management in hepatic GSD. The role of lipids has not been systematically assessed and the current guidelines do not provide clear indications for their use.^2^, ^3^, ^4^, ^5^, ^6^ This systematic literature study and retrospective international multi‐centre cohort study presents that a high fat diet could be considered in paediatric GSDIII patients with cardiomyopathy. The significant reduction in blood CK concentrations and subjective improvement in muscle strength reported in GSDIII patients necessitates further quantification of the effect of a high fat diet on muscle quality and function. Also, liver function, morphology, and growth should be carefully monitored under a high fat regimen given the potential impact on underlying liver disease.

Before discussing the results, some methodological issues need to be addressed. The analysis and interpretation of the data were hampered by large variation in age, dietary intervention (eg, lipid amount, high fat diet alone or together with lipid supplementation), duration of intervention, and outcome parameters. Initially, this study was set up to describe all hepatic GSD types. Most of the data on GSDI and GSDVI were limited and/or historical,^10^, ^12^, ^15^, ^16^, ^18^, ^23^ whereas metabolic control has improved with increasing knowledge on dietary management/glycaemic control and the introduction of management guidelines, as demonstrated for GSDIa patients.^24^ Therefore, in this article, we only included data from GSDIII patients. The published cases presented in this study (*n* = 14) were retrieved from case reports or small cohort studies (describing less than five patients); these data were potentially affected by selection and publication bias. Also, the possible beneficial role of a more compliant dietary scheme during dietary intervention should be considered. Finally, ascertainment bias extends to healthcare professionals attending a GSD conference.

The main indications to start with a dietary lipid manipulation in GSDIII patients were cardiomyopathy, skeletal myopathy or a combination of both. Lipids became the main macronutrient in GSDIII patients at the expense of carbohydrates. Interestingly, cardiac hypertrophy, as quantified by IVSd Z‐scores, decreased only in paediatric GSDIIIa patients. We hypothesize that an early switch to high fat diet can reverse—or at least decrease—the cardiac glycogen storage. Moreover, results showed decreased CK concentrations in 89% of GSDIII patients in accordance with literature^9^, ^11^, ^13^ and improved subjective strength in most of the patients. Increased blood CK concentrations reflect muscle damage which may partially be influenced by exercise. Whether the beneficial effect of a high fat diet on CK concentrations is caused by a lower carbohydrate intake—and thus less accumulation of abnormal glycogen in muscle tissue—or due to the properties of fat to supply alternative energy substrate for muscle remains to be investigated. Notably, most of the GSDIII patients included in the present study received a combination of a high fat and high protein diet. Therefore, these changes in macronutrient composition could also partly account for the beneficial effect on cardiomyopathy and CK concentrations. Nevertheless, protein intake was comparable before and after intervention in GSDIII patients in the present study (Figure 1A).

The development of chronic liver disease is an important concern in ageing GSDIII patients. Although the prevalence of hepatocellular carcinoma was low in the International Study on GSDIII,^25^ severe and progressive liver fibrosis has been described at early ages.^26^ Only one publication describing high fat diet in two GSDIIIa patients documented data on liver transaminases (cases 4 and 5; [9]) Interestingly, we found that ALT concentrations increased in 44% (4/9) of paediatric GSDIII patients, but decreased in all adult GSDIII patients. After dietary lipid manipulation, the concomitant decrease in carbohydrate intake would theoretically lead to less glycogen accumulation in the liver. It remains speculative if these age‐specific effects are part of the natural history or influenced by dietary lipid manipulations. However, under these circumstances, careful monitoring and follow‐up is warranted for liver complications such as hepatosteatosis, liver inflammation, and hepatocellular carcinoma.^27^

Side effects were reported in two patients, consisting in isolated (and mostly mild) hypoglycaemia, an intrinsic symptom in GSD patients.^28^ ‘Side effects’ were not a specific parameter in our data table, and therefore the side effects reported in this study could be an underrepresentation. Previously mentioned concerns regarding MCT in GSD patients are the unknown consequence towards the elongation of fatty acids or gluconeogenesis pathway.^14^ Increased triglycerides concentrations after introduction of MCT have been reported in GSDIII patients.^29^ However, in the present study, the majority of GSDIII patients received a high fat diet rather than MCT supplementation or replacement. As high fat diets have been associated with an increased risk of osteoporosis^30^ combined with the reduced bone mineral density in GSDIII patients^31^ the long‐term effect of dietary lipid manipulations on bone status should be carefully monitored.

Recommendations for future dietary intervention studies and follow‐up of GSDIII patients who start with a high fat diet are summarised in Supplementary File S5. The present study also provides insight in important outcome parameters when assessing the effect of a dietary intervention in hepatic GSD patients. Several additional outcome measures are proposed including muscle,^32^, ^33^, ^34^ bone,^31^ mitochondrial^12^, ^35^ and enzymatic^36^ markers. Prospective, long‐term follow‐up studies are warranted to confirm efficacy and safety of dietary lipid manipulations in the international GSDIII and further hepatic GSD cohort.

## CONFLICT OF INTEREST

A.R received a travel grant from Nestlè Vitaflo to present the results of the study at the International GSD Conference in 2019 in Brazil.

## AUTHOR CONTRIBUTIONS

A.R. and I.J.H. were involved in study design, data collection, data analysis, and wrote the first and final manuscript. V.B.B. and I.J.H. performed the literature search. T.G.J.D. initiated this project, was involved in study design and critically reviewed the versions of the manuscript. All other authors contributed to data collection and revised the manuscript for important intellectual content. All authors approved the final manuscript as submitted and agreed to be accountable for all aspects of the work. All authors confirm the absence of previous similar or simultaneous publications.

## Supporting information


**Supplementary File S1** PRISMA flowchart of search strategy.

**Supplementary File S2** Table published cases.

**Supplementary File S3** Table unpublished cases.

**Supplementary File S4** Individual percentual changes in laboratory parameters of metabolic control for all GSDIII patients.

**Supplementary File S5** Recommendations for clinical follow‐up of dietary lipid manipulations in patients with glycogen storage diseases type III.

## Data Availability

The datasets generated for this study are available on request to the corresponding author.
