# Supplementary material for: Dietary lipids in glycogen storage disease type III: A systematic literature study, case studies, and future recommendations
Source: J Inherit Metab Dis. 2020 Feb 26;43(4):770–7. doi: 10.1002/jimd.12224 (PMC7383479; doi:10.1002/jimd.12224)
Supplement: Supplementary file 1 — Supplementary File S1 PRISMA flowchart of search strategy. [file JIMD-43-770-s001.pdf]

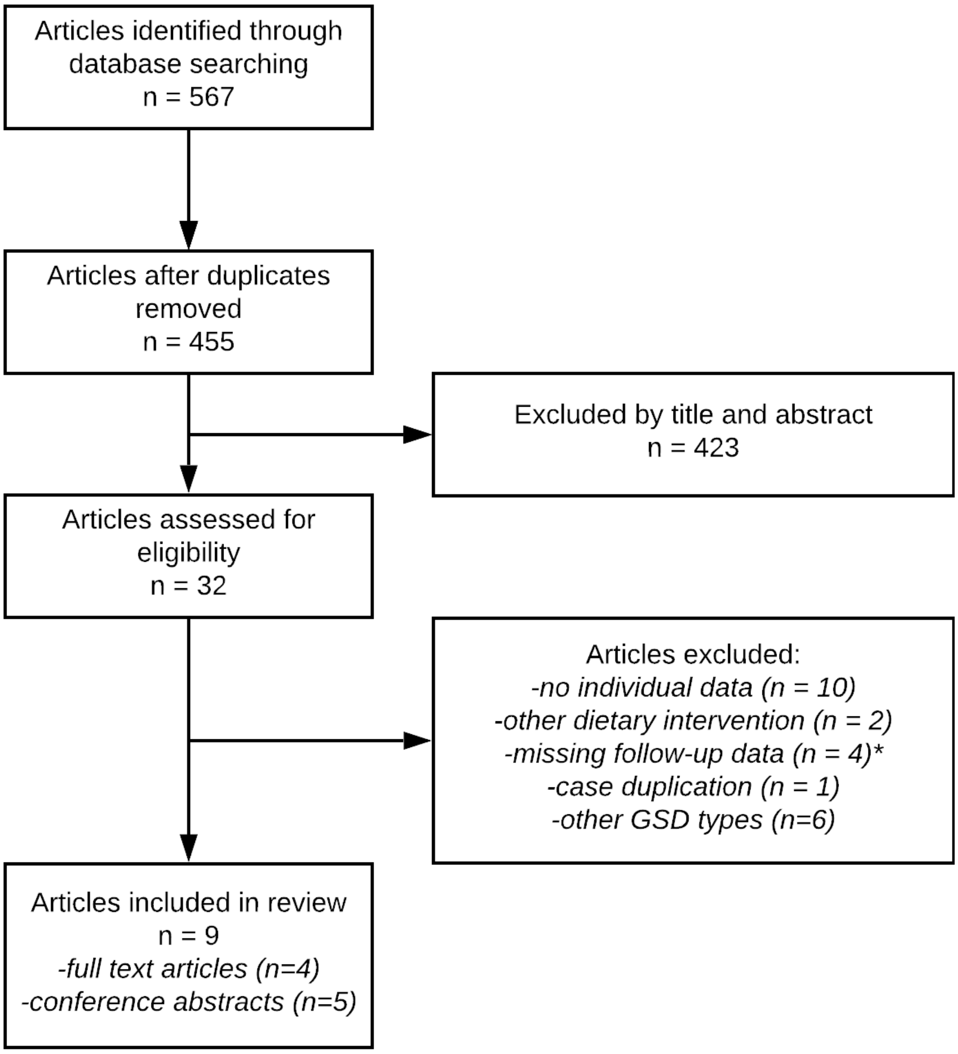

Supplementary File A. Prisma flowchart of search strategy. PubMed and Embase were searched using both MeSH terms and free text: a. PubMed search: ‘("Glycogen Storage Disease"[Mesh] OR glycogen storage[tiab] OR glycogenos*[tiab]) AND ("Ketogenic Diet"[Mesh] OR "Diet, Carbohydrate-Restricted"[Mesh] OR ((fat[tiab] OR fatty*[tiab] OR oil*[tiab] OR atkins[tiab] OR ketogen*[tiab]) AND (diet[tiab] OR diets[tiab] OR dietary[tiab] OR dieting[tiab])) OR "triheptanoin" [Supplementary Concept] OR "Triglycerides"[Mesh] OR "Dietary Fats"[Mesh] OR "Fish Oils"[Mesh] OR medium chain triglycerid*[tiab] OR MCT[tiab] OR triheptanoin*[tiab] OR omega-3-fatty acid*[tiab] OR fish oil*[tiab]) NOT (("Animals"[Mesh] NOT "Humans"[Mesh]) OR animal*[ti] OR rat[ti] OR rats[ti] OR mouse[ti] OR mice[ti]); b. Embase search: ('glycogen storage disease'/exp OR (‘glycogen storage’ OR glycogenos*):ab,ti) AND ('ketogenic diet'/exp OR 'low carbohydrate diet'/exp OR ((fat OR fatty* OR oil* OR atkins OR ketogen*) AND (diet OR diets OR dietary OR dieting)):ab,ti OR 'triheptanoin'/exp OR 'triacylglycerol'/exp OR 'fat intake'/de OR 'fish oil'/exp OR (‘medium chain triglycerid*’ OR MCT OR triheptanoin* OR ‘omega-3-fatty acid*’ OR ‘fish oil*’):ab,ti) NOT ((('animal'/exp OR 'nonhuman'/exp) NOT 'human'/exp) OR (animal* OR rat OR rats OR mouse OR mice):ti). The search was conducted on the 31th of December 2018. The PubMed search revealed 179 articles whereas the Embase search resulted in 388 articles. After the duplicate check a total of 455 articles could be included for the search strategy. *From one of these cases missing data were collected during the retrospective study part; this case was included as unpublished case (case 21) in Supplementary File C.
