# Supplementary material for: Dietary lipids in glycogen storage disease type III: A systematic literature study, case studies, and future recommendations
Source: J Inherit Metab Dis. 2020 Feb 26;43(4):770–7. doi: 10.1002/jimd.12224 (PMC7383479; doi:10.1002/jimd.12224)
Supplement: Supplementary file 4 — Supplementary File S4 Individual percentual changes in laboratory parameters of metabolic control for all GSDIII patients. [file JIMD-43-770-s004.pdf]

	Δ Glucose (%)	Δ Insulin (%)	Δ Ketones (%)	Δ Total Cholesterol (%)	Δ Triglycerides (%)	Δ AST (%)	Δ ALT (%)	Δ CK (%)
P1	-	-	[+]	-	-	-	-	-
P2	-	-	[+]	-	-	-	-	-
P3	-	-	-	-	-	-	-	-31
P4	+6	-	-	+24	-1	-21	-6	-88
P5	+7	-	-	-9	+30	-71	-37	-51
P8	-	-	+2800	-	+39	-	-	-77
P9	-	-	+6000	-	-	-	-	-27
P10	-	-	-	-	-	-	-	-
P11	-	-	-	-	-	-	-	-
P12	-16	-55	+1000	0	0	0	0	-^
P13	-	-	-	0#	0	-	-	-
P14	-	-	-	-[+]	-	-	-	-
P15*	+28	-	+463	+33	+26	-25	-22	-33
P16*	+24	-	+2600	-12	-18	-53	-34	-41
P17	+7	-	+250	-5	-20	+13	10	-38
P18	-16	-35	+450	+9	+41	+0	+8	-65
P19	-4	-	-	+29	-11	+179	+61	-20
P20*	-2	0	-	-3	+4	-	-	-65
P21*	-6	-32	-	+7	-42	-60	-68	-66
P22	+12	-	-	-17	0	-44	-63	-73
P23	+9	-	-	+9	+22	+15	+39	-31
P24	0	-	-	+9	+93	-19	-19	-22
P25	+21	-	-	+22	+42	+304	+83	+32"
P26	+2	-	-	+915	-10	+27	+42	0"
P27	+4	0	-	+31	0	-7	-13	-36
P28	+23	+580	-	+12	+85	-9	+10	-45
Stable (%)	***59***	***33***	***0***	***50***	***37***	***29***	***31***	***5***
Increased (%)	***29***	***17***	***100***	***39***	***42***	***29***	***31***	***5***
Decreased (%)	***12***	***50***	***0***	***11***	***21***	***41***	***38***	***90***

* high fat diet + MCT supplementation
[+] increased. No raw data available
increased after MCT supplementation
^ decreased. No raw data available
" within the reference range
