# Supplementary material for: Dietary lipids in glycogen storage disease type III: A systematic literature study, case studies, and future recommendations
Source: J Inherit Metab Dis. 2020 Feb 26;43(4):770–7. doi: 10.1002/jimd.12224 (PMC7383479; doi:10.1002/jimd.12224)
Supplement: Supplementary file 5 — Supplementary File S5 Recommendations for clinical follow‐up of dietary lipid manipulations in patients with glycogen storage diseases type III. [file JIMD-43-770-s005.pdf]

Recommendations for clinical follow-up of dietary lipid manipulations in patients with glycogen storage diseases type III.

Based on: 'Dietary lipids in glycogen storage disease type III: a systematic literature study, case studies and future recommendations.'

Introduction

Prospectively designed dietary intervention studies are strongly needed to strengthen our knowledge on dietary management in hepatic GSD. With this recommendations document we aim to provide guidance to clinicians and researchers in the field of metabolic disease who intend to study a dietary lipid manipulation, either in clinical management or in the setting of a clinical trial.

Target audience

The present recommendation document is addressed to all health care professionals (physicians and dieticians) who take care of hepatic GSD patients.

Disclaimer

Recommendations are derived from retrospective data collection. Recommendations only refer to dietary lipid manipulations in GSD type IIIa. However, general principles provided here could help arranging a dietary lipid manipulation in all hepatic GSD.

To date, recommendations on dietary management from international management guidelines are still the key in management in hepatic GSD patients.

Index

A – General study recommendations

B – Recommendation sheet lipid manipulation in GSDIIIa patients

A- General study recommendations

I. Patient selection

1) Reasons to start dietary lipid manipulation

Development of cardiomyopathy and/or muscle weakness despite optimal dietary regimen[1].

2) Rationale to start dietary lipid manipulation

Reverse/improve cardiomyopathy and/or myopathy

3) Check contra-indications

-Liver and/or kidney dysfunction

-Osteoporosis

-Current pregnancy, or breastfeeding

-Diabetes mellitus (excluding isolated insulin-resistance)

II. Dietary intervention

- Interventions should be standardized. The amount of fat should be uniform (e.g. high-fat diet, ketogenic diet) as well as the type of fat administered (e.g. high-fat only, high-fat + MCT) and duration of the supplementation.

- Three-day food diaries are recommended to study dietary compliance and analyze exact distributions of macronutrients.

- Amount and duration herein suggested are based on the results of the present study (median value among patients showing beneficial effect)

[1] Guidelines recommend a minimal protein intake of 3 grams per kilogram bodyweight in pediatric GSDIIIa patients.

III. Outcome measures

Outcome measures should be uniform and blood samples should be taken under similar conditions (i.e. specific number of hours after meal and/or specific time during the day). Standard outcome markers should be assessed to make future studies comparable. Taking into account the results of the present study, specific markers are suggested on the next pages.

Improvement should be defined if:

- [CK] decreased by 10% (or more) or normalized
- IVSd Z-scores decreased or normalized

IV. Recommendations on safety

In compliance with Good Clinical Practice (GCP) all adverse events should carefully be assessed and documented.

Possible adverse events:

-Hypoglycemia

-Gastrointestinal symptoms

B- Lipid manipulation in GSDIIIa patients

Dietary intervention: high fat diet

Amount: lipids 60% of daily E-% (children 6 g/kg/day, adults 1.7 g/kg/day)

Minimal duration of intervention: 24 months [range: 3 – 60]

Outcome measures:

Clinical markers: height SDS, weight SDS, BMI, clinical picture (e.g. fatigue, exercise intolerance, dyspnea, muscle strength), comorbidities, QoL questionnaire, International physical activity questionnaire.

Biochemical markers:

- Blood glucose homeostasis: home site continuous glucose monitoring. Number of hypoglycemia (n), mean [range] glucose concentration, percentage of the day [glucose] < 4.0 mmol/L, percentage of the day [glucose] > 8.0 mmol/L. Mean morning ketone concentrations (mmol/L) as assessed with handheld device.

-Blood makers: beta-hydroxybutyrate, acetoacetate, triglycerides, total cholesterol, HDL, LDL, FFA, insulin, AST, ALT, CK, CK-MB, NT-proBNP, TnT, calcium, phosphorus, alkaline phosphatase, parathyroid hormone, calcitonin, osteocalcin, vitamin D, prealbumin, creatinine, estimated glomerular filtration rate, vitamins*, minerals*

- urine: proteinuria, microalbuminuria

- metabolic investigations: plasma acylcarnitines, plasma biotinidase, urine organic acids, urine glucose tetrasaccharide

Imaging markers: liver ultrasound (liver size in cm, liver longitudinal diameter), cardiac ultrasound (IVS thickness, SF, outflow obstruction, diastolic function parameters, left ventricular mass), muscle ultrasound, bone mineral density (DXA), liver/heart/muscle MRI*

Muscle markers: six-minute walking test, muscle ultrasound (muscle density for all muscle groups), dynamometry (strength Z-scores according to references) *

Dietary markers: diet composition (total Kcal/day, E-% and exact amount (g/kg/day) for each macronutrient. Dietary compliance; three-day food diary.

Frequency of follow-up:

Check clinical, blood, and dietary markers monthly for the first 3 months. According to individual outcomes frequency of follow-up can be expanded to every 6 months. Specific metabolic investigations, muscle markers and imaging measures should be at least assessed at the beginning and at the end of the intervention.

* consider
